# Evaluation of dose-efficacy of sorafenib and effect of transarterial chemoembolization in hepatocellular carcinoma patients: a retrospective study

**DOI:** 10.1186/s12876-016-0464-x

**Published:** 2016-04-27

**Authors:** Wang-De Hsiao, Cheng-Yuan Peng, Po-Heng Chuang, Hsueh-Chou Lai, Ken-Sheng Cheng, Jen-Wei Chou, Yang-Yuan Chen, Cheng-Ju Yu, Chun-Lung Feng, Wen-Pang Su, Sheng-Hung Chen, Jung-Ta Kao

**Affiliations:** School of Medicine, China Medical University, Taichung, Taiwan; Division of Hepato-Gastroenterology, Department of Internal Medicine, China Medical University Hospital, No. 2, Yuh-Der Road, Taichung, 404 Taiwan

**Keywords:** Dose-efficacy, Sorafenib, TACE, Hepatocellular carcinoma patient

## Abstract

**Background:**

Transarterial chemoembolization (TACE) and sorafenib are the therapeutic standard for intermediate and advanced stage hepatocellular carcinoma (HCC) patients respectively. High costs with adverse events (AE) of sorafenib might limit sorafenib dosage, further affecting therapeutic response. To attain greatest benefit, we evaluated the efficacy of different doses and effect of TACE during and after sorafenib discontinuation in patients representing Child-Pugh Classification Class A with venous or extra-hepatic invasion.

**Methods:**

A total 156 patients met the criteria and were divided into Groups I (*n* = 52) accepting 800 mg/day; II (*n* = 58) accepting 800 mg/day and reduced to 400 mg/day owing to AE; and III (*n* = 46) accepting 400 mg/day. TACE was performed during and after sorafenib discontinuation and therapeutic response bimonthly to four-monthly was rated thereafter.

**Results:**

Median duration of sorafenib treatment and patients’ survival were 4.00 ± 0.45 and 7.50 ± 1.44 months in all cases; 2.50 ± 0.90 and 5.00 ± 1.10 months in Group I; 5.50 ± 1.27 and 16.50 ± 1.86 months in Group II; 4.00 ± 0.94 and 6.50 ± 2.49 months in Group III. Group II presented the best response and survival benefit (*p* = 0.010 and *p* = 0.011 respectively). Child-Pugh Classification score 5 (Hazard Ratio = 0.492, *p* = 0.049), absent AE (3.423, *p* = 0.015), tumor numbers ≤ 3 (0.313, *p* = 0.009), sorafenib duration ≤ 1 cycle (3.694, *p* = 0.004), and absent TACE (3.197, *p* = 0.008) significantly correlated with patient survival. TACE benefit appeared in separate and total cases during (*p* = 0.002, *p* = 0.595, *p* = 0.074, *p* = 0.002 respectively) and after discontinuation of sorafenib administration (*p* = 0.001, *p* = 0.034, *p* = 0.647, *p* = 0.001 respectively).

**Conclusions:**

Low-dosage sorafenib not only appeared tolerable and lowered economic pressure but also provided satisfactory results. TACE benefited patient’s survival during and after sorafenib discontinuation.

## Background

Worldwide, more than 711,000 new hepatocellular carcinoma (HCC) patients are diagnosed annually; 679,000 eventually die [1]. Various diagnostic and therapeutic modalities have been applied in clinical scenarios [2], with over 50 % of HCC cases showing unresectable or un-embolized condition [3]. Only palliative options are available due to limitations of vascular invasion or extra-hepatic metastases [3, 4].

Sorafenib (Nexavar, Bayer HealthCare Pharmaceuticals-Onyx Pharmaceuticals) inhibits proliferation and angiogenesis in tumors, promoting apoptosis [5, 6]. Anti-angiogenic function is via inhibition of VEGFR-2-PDGFR- and Raf-kinase properties [5–7], signaling pathways identified as a close rationale in HCC study and providing survival benefits in advanced HCC [Barcelona Clinic Liver Cancer (BCLC) stage C] [7–12]. Limitations affect patients treated with sorafenib in clinical scenarios: e.g., high cost raising economic pressure [13], while severe adverse events (AE) (26–88 %) might limit sorafenib dosage and impair therapeutic response, as well as high tumor recurrence with single agent [8, 9, 14, 15]. Therefore, combination therapy provided lesser dose of sorafenib to obtain better response for both in vitro and in vivo models [16]. In clinical settings, transarterial chemoembolization (TACE) is the current standard therapy for intermediate stage HCC (BCLC stage B) and an earlier study indicated combination of TACE with sorafenib as being more effective than TACE or sorafenib monotherapy for unresectable HCC [17]; but no data is reported on the exploration of the effect of different sorafenib dosage with subsequent TACE during or after discontinued sorafenib.

To attain the greatest benefit in clinical scenarios, we assessed two points in this study. First, investigating the relation of different therapeutic doses (initially 400 or 800 mg per day) with efficacy in unresectable HCC patients with compensated liver disease (Child-Pugh Classification Class A [18]) and venous invasion or extra-hepatic metastases. Furthermore, evaluating the effect of TACE during and after sorafenib discontinuation. We envisioned this study could afford useful references for clinical settings.

## Methods

### Patients

**Fig. 1:**
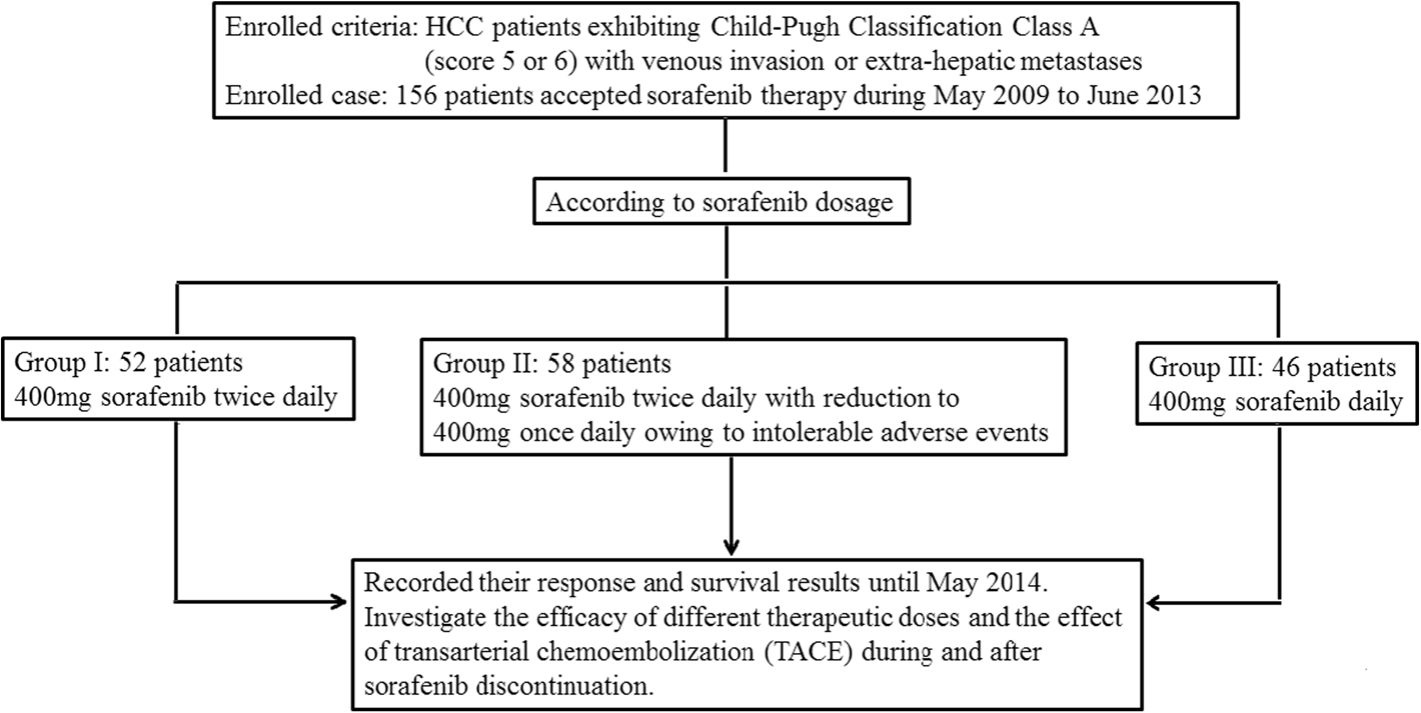
Flow diagram showing the initial therapeutic dose and study aims

According to the standard of Taiwan's National Health Insurance Agency, sorafenib was approved for HCC patients exhibiting Child-Pugh Classification Class A (score 5 or 6) with venous invasion or extra-hepatic metastases. Excluding HCC patients not up to the standard during the period of May 2009 to June 2013, 156 cases (HCC-total group) met the criteria and accepted sorafenib therapy at China Medical University Hospital, Taichung, Taiwan; and their response and survival results were recorded until May 2014. After reviewing their therapeutic dosage between May 2009 and June 2013, patients were divided into three groups: Group I for patients accepting 400 mg sorafenib twice daily; Group II for patients accepting initially 400 mg sorafenib twice daily with reduction to 400 mg once daily owing to intolerable AE; and Group III for patients accepting 400 mg sorafenib daily (Fig. 1). Dose reduction in Group II depended on their tolerance to sorafenib inducing AE (hand-foot skin reaction, uncontrollable hypertension, or diarrhea) rated by the National Cancer Institute (NCI) Common Terminology Criteria for Adverse Events (CTCAE) [19]. The management of AE included corticosteroid cream and painkiller pill for hand-foot skin reaction, anti-hypertension agent for hypertensive patients, as well as loperamide for diarrhea.

### Evaluation of therapeutic response during sorafenib therapy

All treated patients were according to the standard of Taiwan's National Health Insurance Agency and hospital protocol. For each visit while undergoing sorafenib treatment, patients accepted detailed history and physical examination every four weeks. In addition, biochemical examination including Child-Pugh Classification score, serum AFP, renal function, and contrast-enhanced tomography (CT) were performed on those beginning sorafenib and followed up bimonthly to 4-month thereafter according to therapeutic dosage (defined as one cycle) to rate therapeutic response by Response Evaluation Criteria in Solid Tumors criteria (RECIST); only those with better response (complete response [CR], partial response [PR], or stable disease [SD]) could continue sorafenib treatment [20].

### Procedure and effect comparisons of transarterial chemoembolization

In our hospital, TACE proceeds via the trans-femoral route, with arteries feeding tumors identified by angiography and then emulsion of lipiodol (10 mL) and adriamycin (20 mg) were injected, followed by embolization with absorbable particles (gelatin foam). After embolization, more angiography was used to assess extent of vascular occlusion and flow in other arteries. Effect of TACE was evaluated during and after discontinuation of sorafenib administration in separate groups, with follow-up time of either patient death or at least six months after sorafenib discontinuation due to poor therapeutic response. Patients treated with TACE were according to the standard of BCLC System and our hospital for intra-hepatic tumor.

### Serological markers and liver biochemical assay methodology

Commercial enzyme immunoassay rated HBV markers (HBsAg, HBeAg, anti-HBe) (AxSYM, Abbott, North Chicago, IL) and anti-HCV antibody (Abbott HCV EIA 2.0; Abbott Laboratories). An autoanalyzer (TBA-30FR, Toshiba, Tokyo, Japan) gauged serum albumin, bilirubin, alpha-fetoprotein (AFP), aspartate transaminase (AST), alanine Transaminase (ALT), alkaline phosphatase (Alk-p), creatinine (Cr), International Normalize Ratio (INR), and hematological count (WBC: white blood cell; Hb, hemoglobin; platelet).

### Statistical analysis

All statistical analyses were performed using SPSS 17.0 (SPSS, Chicago, USA). Baseline data were expressed as mean ± standard deviation, with correlation between continuous variables assessed by Student *t*-test or Fisher exact test. In univariate survival analysis, the median survival times were calculated according to the Kaplan-Meier method. All variables significant in univariate analysis were entered into the multivariate model according to Cox proportional hazard regression. All statistical tests were two-tailed, *P*-value ≤ 0.05 defined as significant.

## Results

### General distribution of overall and separate hepatocellular carcinoma groups

Table 1 shows baseline characteristics of 156 HCC cases. Median durations of sorafenib treatment and patients’ survival were 4.00 ± 0.45 months (95 % CI 3.13–4.87 months) and 7.50 ± 1.44 months (95 % CI 4.67–10.33 months) respectively. There were 57 patients without and 99 with AE: e.g., hand-foot skin reaction (*n* = 53 cases; 45 in grade 1 and 2; 8 in grade 3), diarrhea (*n* = 24 cases; 19 in grade 1 and 2; 5 in grade 3), hypertension (*n* = 5 cases; 4 in grade 1 and 2; 1 in grade 3), combination of hand-foot skin reaction and diarrhea (*n* = 17; 14 in grade 1 and 2; 3 in grade 3). No grade 4 or mortality case was induced from AE. According to therapeutic dosage, 52 cases belonged to Group I, 56 cases belonged to Group II, and 46 cases belonged to Group III (Fig. 1). No significant difference between the three groups besides lower albumin (3.68 ± 0.51 versus 4.05 ± 0.42 g/dL, *p* < 0.001) with advanced age (64.50 ± 12.02 versus 58.75 ± 13.33 years, *p* = 0.028) in Group III than Group I. In separate groups, median durations of sorafenib treatment and survival were 2.50 ± 0.90 months (95 % CI 0.73–4.27 months) and 5.00 ± 1.10 months (95 % CI 2.85–7.16 months) in Group I; 5.50 ± 1.27 months (95 % CI 3.01–7.99 months) and 16.50 ± 1.86 months (95 % CI 12.86–20.14 months) in Group II; 4.00 ± 0.94 months (95 % CI 2.16–5.84 months) and 6.50 ± 2.49 months (95 % CI 1.63–11.37 months) in Group III respectively.

**Table 1:** Baseline characteristics of total and separate hepatocellular carcinoma groups treated with sorafenib

Demographics	Total cases (*n* = 156)	Group I (*n* = 52)	Group II (*n* = 58)	Group III (*n* = 46)
Age (yrs) (range)	61.04 ± 12.76 (32.00–88.00)	58.75 ± 13.33 (32.0–84.0)	60.36 ± 12.45 (32.0–88.0)	64.50 ± 12.02 (40.0–86.0)
Sex (Male) (%)	127 (81.41)	46 (88.5)	52 (89.66)	29 (63.04)
BMI (kgs/m^2^) (range)	22.69 ± 3.88(14.10–34.82)	23.05 ± 3.71 (15.24–34.82)	21.92 ± 3.48 (14.10–32.86)	23.26 ± 4.43 (15.56–33.75)
Cirrhosis (+) (%)	120 (76.92)	32 (61.5)	50 (86.21)	38 (82.61)
CPC, score 5 versus 6 (%)	81 (51.92) versus 75 (48.08)	30 (57.7) versus 22 (42.3)	31 (53.45) versus 27 (46.55)	20 (43.48) versus 26 (56.52)
Biochemical values				
Albumin (g/dL) (range)	3.88 ± 0.50 (2.80–5.10)	4.05 ± 0.42 (3.20–4.80)	3.88 ± 0.51 (2.80–5.10)	3.68 ± 0.51 (2.80–4.70)
Bilirubin (mg/dL) (range)	1.02 ± 0.46 (0.19–2.41)	0.99 ± 0.41 (0.22–1.99)	1.03 ± 0.48 (0.19–2.29)	1.05 ± 0.51 (0.38–2.41)
INR (range)	1.14 ± 0.13 (0.89–1.63)	1.13 ± 0.12 (0.90–1.63)	1.15 ± 0.13 (0.90–1.48)	1.15 ± 0.15 (0.89–1.63)
AST (IU/L) (range)	92.45 ± 105.98 (22.00–805.00)	96.20 ± 102.12 (24.00–580.00)	95.07 ± 136.90 (22.00–805.00)	84.84 ± 53.33 (23.00–237.00)
ALT (IU/L) (range)	66.54 ± 102.12 (9.00–1156.00)	65.73 ± 48.32 (15.00–251.00)	76.86 ± 157.43 (9.00–1156.00)	54.43 ± 39.91 (13.00–202.00)
ALK-P (IU/L) (range)	127.76 ± 93.34 (43.00–680.00)	124.84 ± 81.74 (50.00–421.00)	113.14 ± 93.05 (45.00–680.00)	155.0 ± 104.82 (43.00–526.00)
AFP (ng/mL) (range)	9000.31 ± 17472.65 (0.91–54001.0)	11769.7 ± 19836.59 (1.64–54001.0)	6663.7 ± 14569.6 (0.91–54001.0)	8815.86 ± 17910.81 (1.30–54001.0)
Cr (mg/dL) (range)	0.93 ± 0.35 (0.21–2.76)	0.90 ± 0.30 (0.40–2.10)	0.94 ± 0.32 (0.27–1.76)	0.95 ± 0.43 (0.21–2.76)
WBC (10^3^/uL) (range)	6.90 ± 3.71 (1.70–22.62)	6.96 ± 3.33 (2.48–17.35)	6.79 ± 3.66 (1.70–19.00)	7.00 ± 4.23 (2.10–22.62)
Hb (gm/dL) (range)	12.48 ± 1.97 (7.90–17.10)	12.63 ± 1.98 (7.90–16.00)	12.64 ± 2.02 (7.90–17.10)	12.11 ± 1.89 (8.10–15.10)
Platelet (10^3^/uL) (range)	171.49 ± 110.79 (16.00–796.00)	170.69 ± 82.45 (16.00–400.00)	177.19 ± 145.22 (44.00–796.0)	165.2 ± 88.18 (23.00–386.00)
Virologic values				
B or C or B + C (+) or NBNC (%)	80 (51.28) or 50 (32.05) or 5 (3.21) or 21 (13.46)	30 (57.7) or 9 (17.3) or 3 (5.8) or 10 (19.2)	33 (56.9) or 18 (31.03) or 1 (1.72) or 6 (10.34)	17 (36.96) or 23 (50.0) or 1 (2.17) or 5(10.87)
Tumor characters				
Tumor size (>5 cm) (%)	74 (47.44)	27 (51.9)	25 (43.1)	22 (47.83)
Tumor number (>3) (%)	91 (58.33)	28 (53.8)	32 (55.17)	31 (67.39)
Intra-hepatic vein (+) (%)	61 (39.10)	23 (44.2)	18 (31.03)	20 (43.48)
Extra-hepatic metastases (%)	75 (48.08)	23 (44.2)	31 (53.45)	21 (45.65)
Mixed type (vein and metastases)	20 (12.82)	6 (11.5)	9 (15.52)	5 (10.87)

*Abbreviations*: *CPC* child-pugh classification, *INR* international normalize ratio, *AST* aspartate transaminase, *ALT* alanine transaminase, *Alk-p* alkaline phosphatase, *GGT* gamma-glutamyltransferase, *AFP* alpha-fetoprotein, *Cr* creatinine, *WBC* white blood cell, *Hb* hemoglobin, *B* hepatitis B virus, *C* hepatitis C virus, *B + C* hepatitis B and C virus, *NBNC* non-hepatitis B or C virus

### Survival analysis in overall and separate groups

In total cases, the significant factors including Child-Pugh Classification score 5 (*p* = 0.015), absent AE (*p* < 0.001), lower ALT (*p* = 0.036), lower AFP (*p* < 0.001), tumor size ≤ 5 cm (*p* = 0.046), tumor numbers ≤ 3 (*p* = 0.001), sorafenib duration ≤ 1 cycle (*p* < 0.001), and absent TACE (*p* = 0.031) affected patient’s survival. Of all significant variables, Child-Pugh Classification score 5 (Hazard Ratio = 0.492, *p* = 0.049), absent AE (Hazard Ratio = 3.423, *p* = 0.015), lower AFP (Hazard Ratio = 0.213, *p* = 0.003), tumor numbers ≤ 3 (Hazard Ratio = 0.313, *p* = 0.009), sorafenib duration ≤ 1 cycle (Hazard Ratio = 3.694, *p* = 0.004), and absent TACE (Hazard Ratio = 3.197, *p* = 0.008) significantly correlated with patient mortality (Table 2). Among separate groups, absent AE (*p* = 0.008), lower AFP (*p* = 0.014), tumor numbers ≤ 3 (*p* = 0.032), and sorafenib duration ≤ 1 cycle (*p* = 0.003) presented significant difference affecting patient’s survival. Of all significant variables, lower AFP (Hazard Ratio = 0.136, *p* = 0.047) and sorafenib duration ≤ 1 cycle (Hazard Ratio = 8.112, *p* = 0.040) played independent roles to predict patient’s mortality in Group I. In Group II, the factors including lower AFP (*p* = 0.043), sorafenib duration ≤ 1 cycle (*p* = 0.002), and absent TACE (*p* = 0.041) presented significant difference affecting patient’s survival. Of all significant variables, sorafenib duration ≤ 1 cycle (Hazard Ratio = 7.080, *p* = 0.014) and absent TACE (Hazard Ratio = 6.742, *p* = 0.022) played independent roles to predict patient’s mortality. In Group III, lower albumin (*p* = 0.048) and tumor numbers ≤ 3 (*p* = 0.023) presented significant difference affecting patient’s survival. Of all significant variables, lower albumin (Hazard Ratio = 5.989, *p* = 0.046) and tumor numbers ≤ 3 (Hazard Ratio = 0.187, *p* = 0.025) played independent roles to predict patient’s mortality.

**Table 2:** Cox regression of mortality in overall hepatocellular carcinoma patients. (*N* = 156)

	Numbers	*P*-value		Hazard Ratio (95 % CI)
		Univariate	Multivariate	
Demographics				
Age (yrs), ≤65 vs. >65	100 vs. 56	0.613		
Gender, Female vs. Male	29 vs. 127	0.863		
BMI (kgs/m^2^), ≤22 (24) vs. >22 (24)	102 vs. 54	0.798		
Average dose (mg/kg), ≤35 vs. >35	97 vs. 59	0.848		
Cirrhosis, (-) vs. (+)	36 vs. 120	0.263		
CPC, score 5 vs. 6	81 vs. 75	0.015*	0.049*	0.492 (0.213–1.137)
AE, (-) vs. (+)	57 vs. 99	<0.001*	0.015*	3.423 (1.274–9.199)
Biochemical values				
Albumin (g/dL), ≤3.5 vs. >3.5	35 vs. 121	0.061		
Bilirubin (mg/dL), ≤1.3 vs. > 1.3	118 vs. 38	0.466		
INR, ≤1.2 vs. >1.2	116 vs. 40	0.321		
AST (IU/L), ≤34 vs. >34	25 vs. 130	0.181		
ALT (IU/L), ≤40 vs. >40	73 vs.83	0.036*		
Alk-p (IU/L), ≤126 vs. >126	87 vs.39	0.051		
AFP (ng/mL), ≤9 vs. > 9	34 vs. 122	<0.001*	0.003*	0.213 (0.078–0.583)
Creatinine (mg/dL), ≤1.3 vs. > 1.3	138 vs. 18	0.188		
WBC (10^3^/dL), ≤105 vs. >105	136 vs.17	0.553		
Hb (gm/dL), ≤12 vs. >12	62 vs.94	0.115		
Platelet (10^3^/uL), ≤130 vs. >130	61 vs. 95	0.207		
Virologic values				
HBV or HCV, (-) vs. (+)	21 vs. 135	0.873		
Tumor characters				
Tumor size, ≤5 vs. >5	82 vs. 74	0.046*		
Tumor numbers, ≤3 vs. >3	65 vs. 91	0.001*	0.009*	0.313 (0.131–0.747)
Intra-hepatic vein, (-) vs. (+)	95 vs. 61	0.661		
Extra-hepatic metastases, (-) vs. (+)	81 vs. 75	0.388		
Mixed type (vein and metastases), (-) vs. (+)	136 vs. 20	0.517		
Therapeutic response				
Sorafenib duration, cycle ≤ 1 vs. >1	75vs. 81	<0.001*	0.004*	3.694 (1.530–8.920)
TACE (-) vs. (+)	89 vs. 67	0.031*	0.008*	3.197 (1.353–7.553)

*Abbreviations*: *AE* adverse event, *AST* aspartate transaminase, *ALT* alanine transaminase, *Alk-p* alkaline phosphatase, *AFP* alpha-fetoprotein, *BMI* body mass index (Cut-off valve: 22 in female and 24 in male), *CPC* child-pugh classification, *Hb* hemoglobin, *B* hepatitis B virus, *C* hepatitis C virus, *INR* international normalize ratio, *NBNC* non-hepatitis B or C virus, *TACE* transarterial chemoembolization, *WBC* white blood cell

^*^A *P*-value below 0.05 is considered statistically significant

### Positive correlation between better sorafenib response and higher survival

**Fig. 2:**
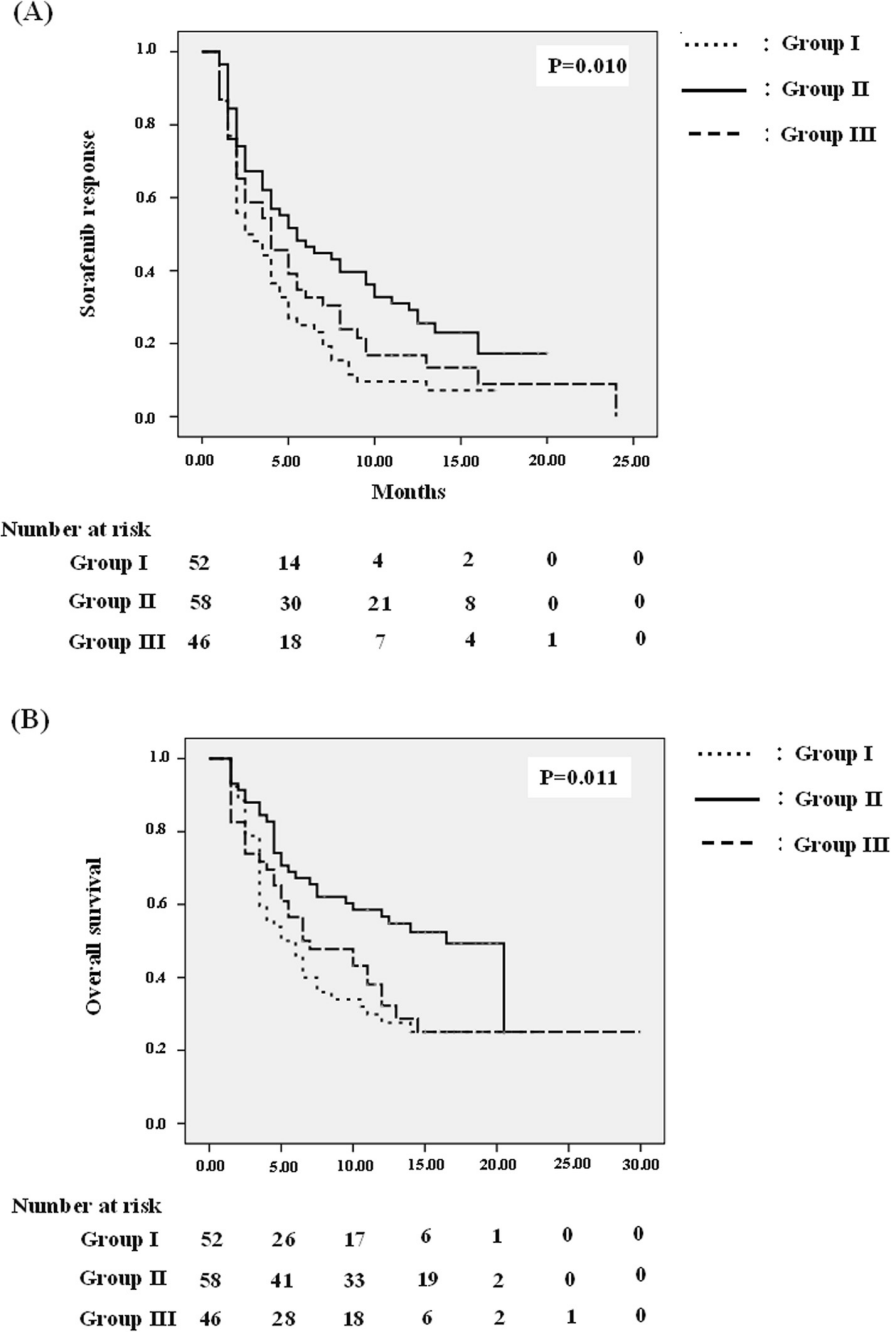
Kaplan-Meier analysis of sorafenib response (**a**) and overall survival (**b**). *P*-value below 0.05 is considered statistically significant

There was a positive correlation between sorafenib duration and survival time (*r* = 0.756, *p* < 0.001). Group II showed the best sorafenib response and overall survival among the three groups (*p* = 0.010 and *p* = 0.011 respectively) (Fig. 2).

### TACE increases survival during and after sorafenib discontinuation

Over the study period, presence or absence of TACE presented significant difference (*p* = 0.031) and significantly correlated with patient mortality (Hazard Ratio = 3.197, *p* = 0.008) (Table 2). During the sorafenib period, patients accepting TACE revealed lower mortality than those without TACE, particularly in total patients (39/57 versus 75/99, *p* = 0.002) and Group I (14/21 versus 28/31, *p* = 0.002) (Table 3). Patients accepting TACE had younger age (57.54 ± 13.00 versus 63.06 ± 12.24 years, *p* = 0.009) than those without TACE among HCC total patients; lower AST (62.48 ± 37.92 versus 119.80 ± 124.85 IU/L, *p* = 0.024), higher albumin (4.20 ± 0.43 versus 3.95 ± 0.39 g/dL, *p* = 0.041), and lower BMI (21.78 ± 3.77 versus 23.91 ± 3.47 kg/m^2^, *p* = 0.041) or higher average dose (37.85 ± 6.89 versus 34.10 ± 4.61 mg/kg, *p* = 0.036) in Group I; lower Cr (0.83 ± 0.21 versus 1.00 ± 0.36 mg/dL, *p* = 0.022) and higher rate of tumor numbers > 3 (16/21 versus 16/37, *p* = 0.027) in Group II; higher BMI (26.35 ± 4.21 versus 22.77 ± 3.77 kg/m^2^, *p* = 0.001), higher Hb (13.04 ± 1.98 versus 11.66 ± 1.70gm/dL, *p* = 0.019), and lower rate of tumor size < 5 cm (3/15 versus 19/31, *p* = 0.012) in Group III. After discontinued sorafenib due to poor response, patients accepting TACE also presented lower mortality than those without TACE particularly in total patients (13/26 versus 67/83, *p* = 0.001) and Groups I (6/10 versus 24/28, *p* = 0.001) with II (4/10 versus 23/30, *p* = 0.034) (Table 3). Patients accepting TACE had lower AST (56.16 ± 27.09 versus 97.35 ± 129.26 IU/L, *p* = 0.008) than those without TACE among HCC total patients; lower virology rate (6/10 versus 28/30, *p* = 0.026) in Group II; lower Alk-p (81.25 ± 18.66 versus 130.67 ± 64.17 IU/L, *p* = 0.009), lower average dose (16.04 ± 2.14 versus 20.69 ± 5.83 mg/kg, *p* = 0.004), and lower cirrhotic rate (3/6 versus 23/25, *p* = 0.038) in Group III. No mortality case was induced by TACE.

**Table 3:** Comparison of mortality in presence or absence of TACE during and after discontinuation of sorafenib administration

	Sorafenib (+) TACE (+) vs. TACE (-)		Sorafenib (-) TACE (+) vs. TACE (-)
Subgroups		Subgroups	
Group I (*n* = 52)	14/21 vs. 28/31 *p* = 0.002*	Group I (*n* = 38)	6/10 vs. 24/28 *p* = 0.001*
Group II (*n* = 58)	13/21 vs. 23/37 *p* = 0.595	Group II (*n* = 40)	4/10 vs. 23/30 *p* = 0.034*
Group III (*n* = 46)	12/15 vs. 24/31 *p* = 0.074	Group III (*n* = 31)	3/6 vs. 20/25 *p* = 0.647
HCC-total Patients (*N* = 156)	39/57 vs. 75/99 *p* = 0.002*	HCC-total Patients (*N* = 109)	13/26 vs. 67/83 *p* = 0.001*

Abbreviations: Group I always accepted 800 mg/day; Group II initially accepted 800 mg/day tapering to 400 mg/day owing to adverse events; Group III always accepted 400 mg/day. *TACE* transarterial chemoembolization

*A *P*-value below 0.05 is considered statistically significant

## Discussion

Sorafenib inhibits proliferation and angiogenesis while promoting apoptosis of tumors [5, 6], and is proven to prolong survival in advanced HCC cases [8, 9]. As in earlier studies [8, 9, 21–23], the clinical factors including superior liver preservation (score 5), lower AFP, or less aggressive tumor condition remained as the crucial roles to reflect patient results in our study (Table 2). Therefore, highly selected patients should be emphasized in initial sorafenib therapy.

Nevertheless, problems remain in clinical practice, including high sorafenib costs that raise economic pressure [13], as well as severe AE rate from 26 to 88 % that might limit sorafenib dosage and further affect therapeutic response [8, 9, 14, 15]. To attain greatest benefit, we analyzed the relationship of different sorafenib doses with efficacy. As in previous studies [8, 9], the presence of AE also played an important role predicting therapeutic response. Importantly, patients could tolerate longer duration (>1 cycles) of obtained therapeutic benefit from sorafenib (Table 2). Furthermore, we found despite Groups II and III presenting poorer baseline characteristics than I including lower albumin (3.68 ± 0.51 g/dL versus 4.05 ± 0.42 g/dL, *p* < 0.001 respectively) and older age (64.5 ± 12.02 versus 58.75 ± 13.33 years, *p* = 0.028 respectively) (Table 1), Groups II and III rather than I still showed better sorafenib response and survival benefit (*p* = 0.010 and *p* = 0.011 respectively) (Fig. 2) as our finding of poorer sorafenib response significantly correlated with higher mortality (*r* = 0.756, *p* < 0.001). This revealed sorafenib, even at lower dosages, could also provide therapeutic benefit; particularly for patients tolerating longer sorafenib duration [24]. Accordingly, this could alleviate economic pressure with tolerable dose for HCC therapy as well as wastage of medical resources.

In clinic, combination of TACE with sorafenib has proven more effective than TACE or sorafenib monotherapy for unresectable HCC [17], but no data has reported on the exploration of the effect of different sorafenib dose with subsequent TACE during or after discontinued sorafenib owing to poor response. In this study, we observed combined TACE promoted patient’s survival during the period with sorafenib, which concurred with a previous finding: the sorafenib/TACE combination shows promise as an effective and tolerable treatment strategy for unresectable patients with intermediate stage/advanced HCC [17]. The benefit of TACE also appeared in patients after discontinued sorafenib, particularly in Group II where lower mortality in TACE cases existed after discontinuation than for those during sorafenib administration (Tables 2 and 3). This possibility could be attributed to AE induced by sorafenib, regarded as an indicator of better therapeutic response presenting enough anti-tumor concentrations [25, 26]. Therefore, TACE showed lower benefit during the period with sorafenib than that without sorafenib. Once tumor inhibition with sorafenib significantly decreased due to poor response and probably induced tumor re-growth, the effect of TACE became obvious. Group III rather than I or II presented insignificant benefit from TACE after discontinued sorafenib, the limited case number probably contributed to the result, and further larger studies need to be adopted in the future.

Although median overall survival in our study (7.50 ± 1.44 months) was shorter than SHARP (10.7 months) [8], better than Asian-Pacific studies (6.5 months) [9] and probably exceeded the SHARP study: 21 patients (4 in Group I, 12 in Group II, 5 in Group III) still accepted sorafenib and 42 cases (10 in Group I, 22 in Group II, 10 in Group III) had survived at end of follow-up time. Additionally, we correlated body mass index (kgs/m^2^) with average sorafenib dose [mg/(kgs/m^2^)] and patient’s survival, with no significant differences in HCC total cases or separate groups (Table 2). Even after excluding TACE cases from the period with sorafenib, higher average dosage also could not promote therapeutic response [31.86 ± 9.54 versus 29.48 ± 9.78 mg/(kgs/m^2^), *p* = 0.246] and prolong survival [31.39 ± 9.73 versus 30.01 ± 9.32 mg/(kgs/m^2^), *p* = 0.581].

## Conclusions

According to our interpretations, low-dosage sorafenib also provided satisfactory therapeutic result, which not only appeared tolerable but also lowered economic pressure and conserved medical resources. Beside the initial variables including the presence of AE, superior liver preservation, lower AFP, less aggressive tumor condition, and longer sorafenib duration benefited patient’s survival, synergic TACE promoted patient’s survival during the period of sorafenib administration and also appeared after sorafenib discontinuation. Our study provided much useful information in unresectable HCC patients with compensated liver disease and venous invasion or extra-hepatic metastases but was limited in the size of cohorts; therefore, further larger studies need to be adopted in the future.

## Abbreviations

AE: adverse events
AFP: alpha-fetoprotein
Alk-p: alkaline phosphatase
ALT: alanine transaminase
AST: aspartate transaminase
CR: complete response
Cr: creatinine
CT: contrast-enhanced tomography
CTCAE: common terminology criteria for adverse events
Hb: hemoglobin
HBV: hepatitis B Virus infection platelet
HCC: hepatocellular carcinoma
HCV: hepatitis C Virus infection
INR: international normalize ratio
NCI: National Cancer Institute
PR: partial response
RECIST: response evaluation criteria in solid tumors criteria
SD: stable disease
TACE: transarterial chemoembolization
WBC: white blood cell

## References

[1] Garcia M, Jemal A, Ward EM, Center MM, Hao Y, Siegel RL, et al. Global Cancer Facts and Figures. 2007. https://www.cancer.org/acs/groups/content/@nho/documents/document/globalfactsandfigures2007rev2p.pdf.

[2] Bruix J, Sherman M (2011). Management of hepatocellular carcinoma: an update. Hepatology.

[3] Forner A, Reig ME, de Lope CR, Bruix J (2010). Current strategy for staging and treatment: BCLC update and future prospects. Semin Liver Dis.

[4] Imamura H, Matsuyama Y, Tanaka E, Ohkubo T, Hasegawa K, Miyagawa S (2003). Risk factors contributing to early and late phase intrahepatic recurrence of hepatocellular carcinoma after hepatectomy. J Hepatol.

[5] Wilhelm SM, Carter C, Tang L, Wilkie D, McNabola A, Rong H (2004). BAY 43-9006 exhibits broad-spectrum oral antitumor activity and targets the RAF/MEK/ERK pathway and receptor tyrosine kinases involved in tumor progression and angiogenesis. Cancer Res.

[6] Chang YS, Adnane J, Trail PA, Levy J, Henderson A, Xue D (2007). Sorafenib (BAY 43-9006) inhibits tumor growth and vascularization and induces tumor apoptosis and hypoxia in RCC xenograft models. Cancer Chemother Pharmacol.

[7] Semela D, Dufour JF (2004). Angiogenesis and hepatocellular carcinoma. J Hepatol.

[8] Llovet JM, Ricci S, Mazzaferro V, Hilgard P, Gane E, Blanc JF (2008). Sorafenib in advanced hepatocellular carcinoma. N Engl J Med.

[9] Cheng AL, Kang YK, Chen Z, Tsao CJ, Qin S, Kim JS (2009). Efficacy and safety of sorafenib in patients in the Asia-Pacific region with advanced hepatocellular carcinoma: a phase III randomised, double-blind, placebo-controlled trial. Lancet Oncol.

[10] Ito Y, Sasaki Y, Horimoto M, Wada S, Tanaka Y, Kasahara A (1998). Activation of mitogen-activated protein kinases/extracellular signal-regulated kinases in human hepatocellular carcinoma. Hepatology.

[11] Omata M, Lesmana LA, Tateishi R, Chen PJ, Lin SM, Yoshida H (2010). Asian Pacific Association for the Study of the Liver consensus recommendations on hepatocellular carcinoma. Hepatol Int.

[12] Calvisi DF, Ladu S, Gorden A, Farina M, Conner EA, Lee JS (2006). Ubiquitous activation of Ras and Jak/Stat pathways in human HCC. Gastroenterology.

[13] Carr BI, Carroll S, Muszbek N, Gondek K (2010). Economic evaluation of sorafenib in unresectable hepatocellular carcinoma. J Gastroenterol Hepatol.

[14] Ogasawara S, Kanai F, Obi S, Sato S, Yamaguchi T, Azemoto R (2011). Safety and tolerance of sorafenib in Japanese patients with advanced hepatocellular carcinoma. Hepatol Int.

[15] Kudo M, Imanaka K, Chida N, Nakachi K, Tak WY, Takayama T (2011). Phase III study of sorafenib after transarterial chemoembolisation in Japanese and Korean patients with unresectable hepatocellular carcinoma. Eur J Cancer.

[16] Alsaied OA, Sangwan V, Banerjee S, Krosch TC, Chugh R, Saluja A (2014). Sorafenib and triptolide as combination therapy for hepatocellular carcinoma. Surgery.

[17] Abdel-Rahman O, Elsayed ZA (2013). Combination Trans Arterial Chemoembolization (TACE) Plus Sorafenib for the Management of Unresectable Hepatocellular Carcinoma: A Systematic Review of the Literature. Dig Dis Sci.

[18] Pugh RN, Murray-Lyon IM, Dawson JL, Pietroni MC, Williams R (1973). Transection of the oesophagus for bleeding oesophageal varices. Br J Surg.

[19] Trotti A, Colevas AD, Setser A, Rusch V, Jaques D, Budach V (2003). CTCAE v3.0: development of a comprehensive grading system for the adverse effects of cancer treatment. Semin Radiat Oncol.

[20] Therasse P, Arbuck SG, Eisenhauer EA, Wanders J, Kaplan RS, Rubinstein L (2000). New guidelines to evaluate the response to treatment in solid tumors. European Organization for Research and Treatment of Cancer, National Cancer Institute of the United States, National Cancer Institute of Canada. J Natl Cancer Inst.

[21] Changchien CS, Chen CL, Yen YH, Wang JH, Hu TH, Lee CM (2008). Analysis of 6381 hepatocellular carcinoma patients in southern Taiwan: prognostic features, treatment outcome, and survival. J Gastroenterol.

[22] Carr BI, Pancoska P, Branch RA (2009). Tumor and liver determinants of prognosis in unresectable hepatocellular carcinoma: a large case cohort study. Hepatol Int.

[23] Bouattour M, Payancé A, Wassermann J (2015). Evaluation of antiangiogenic efficacy in advanced hepatocellular carcinoma: Biomarkers and functional imaging. World J Hepatol.

[24] Ogasawara S, Chiba T, Ooka Y, Kanogawa N, Motoyama T, Suzuki E (2014). Is intra-patient sorafenib dose re-escalation safe and tolerable in patients with advanced hepatocellular carcinoma?. Int J Clin Oncol.

[25] Vincenzi B, Santini D, Russo A, Addeo R, Giuliani F, Montella L (2010). Early Skin Toxicity as a Predictive Factor for Tumor Control in Hepatocellular Carcinoma Patients Treated with Sorafenib. Oncologist.

[26] Arrondeau J, Mir O, Boudou-Rouquette P, Coriat R, Ropert S, Dumas G (2012). Sorafenib exposure decreases over time in patients with hepatocellular carcinoma. Invest New Drugs.

